# Evaluating of neonatal early onset sepsis through lactate and base excess monitoring

**DOI:** 10.1038/s41598-023-41776-0

**Published:** 2023-09-08

**Authors:** Aslan Yilmaz, Nesrin Kaya, Ilker Gonen, Abdulkerim Uygur, Yildiz Perk, Mehmet Vural

**Affiliations:** 1 Department of Neonatology, Cerrahpasa Faculty of Medicine, Istanbul University-Cerrahpasa, 34098 Kocamustafapasa, Fatih, Istanbul Turkey; 2 Department of Neonatology, Kanuni Sultan Süleyman Training and Research Hospital, University of Health Sciences, Istanbul, Turkey; 3 Department of Public Health, Cerrahpasa Faculty of Medicine, Istanbul University-Cerrahpasa, 34098 Kocamustafapasa, Fatih, Istanbul Turkey

**Keywords:** Biochemistry, Biomarkers

## Abstract

Early-onset sepsis (EOS) is one of the leading causes of neonatal death and morbidity worldwide and timely initiation of antibiotic therapy is, therefore, of paramount importance. This study aimed to evaluate the predictive effect of lactate and base excess (BE) values in the cord arterial blood gas and the 6th hour of life venous blood gas analysis on clinical sepsis in newborns. This is a cohort case–control study. In this study, 104 cases were divided into clinical and suspected sepsis groups according to the evaluation at the 24th hour after delivery. Lactate and BE values were evaluated in the cord arterial blood gas analysis (ABGA) and at the postnatal 6th-hour venous blood gas. The cord ABGA and postnatal 6th-hour results were compared in the clinical and suspected sepsis groups. Clinical sepsis was found to be associated with a lactate value above 2 mMol/L at postnatal 6th-hour venous blood gas (*p* = 0.041). This association was the highest when the clinical sepsis group's postnatal 6th-hour lactate cut-off value was determined as 3.38 mMol/L (sensitivity 57.9% and specificity 68.5%) (*p* = 0.032). However, no association was found between clinical sepsis diagnosis and venous BE's value in cord ABGA at the postnatal 6th hour. We found that a venous lactate value above 3.38 mMol/L at the postnatal 6th hour was the cut-off value that could indicate early-onset clinical sepsis. However, none of the biomarkers used in diagnosing EOS can accurately show all cases.

## Introduction

Neonatal sepsis is the leading cause of global death in children under 5 years^1^. Proven neonatal early-onset sepsis (EOS) has mortality rates of up to 30% in high-income and 60% in low-income countries^2^. EOS is defined as the onset of sepsis within 72 h after birth. EOS risk factors were reported as group B streptococcal colonization, chorioamnionitis, prelabor rupture of membranes (at least 18 h before the onset of delivery), maternal fever, and gestational week^3,4^.

Rapid diagnosis and treatment of neonatal EOS are crucial to preventing morbidity and mortality. Clinical findings are often vague and non-specific, and commonly used biomarkers have low predictive values for early sepsis^5^. Although many biomarkers have been tried to predict sepsis, no definitive diagnostic marker has yet been found^6^. Another method to increase the accuracy of EOS diagnosis is the European Medicines Agency (EMA) scoring, which has been developed using a combination of clinical and laboratory findings and is frequently applied in neonatal units. This scoring system criteria include lactate and BE values^7^. pH indicates metabolic acidosis and results from the balance between lactate, which tends to lower pH and BE, which stabilizes it^8^. Mild metabolic acidosis detected in cord blood gas of term newborns is associated with morbidity^9^. In addition, recent studies show a relationship between lactate level and neonatal sepsis and morbidity^10,11^.

This study aimed to evaluate the predictive effect of lactate and BE analysis in cord artery blood gas (ABGA) and 6th-hour venous blood gas on clinical sepsis and suspected sepsis.

## Materials and methods

**Figure 1:**
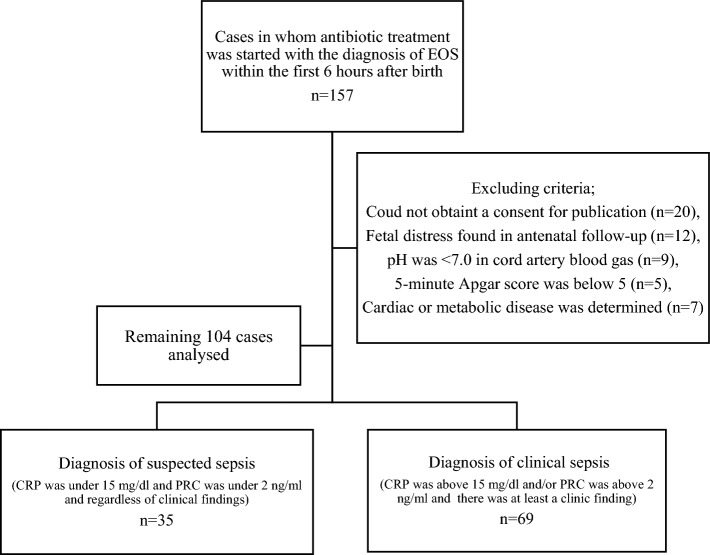
Study diagram. *EOS* Early-onset sepsis, *ABGA* Arterial blood gas analysis, *BE* Base excess, *CRP* C-reactive protein, *PCT* Procalcitonin, *WBC* White blood cell.

The study was conducted Cerrahpasa Faculty of Medicine, Istanbul University and Kanuni Sultan Suleiman Training and Research Hospital in Istanbul between January 1, 2019, and December 31, 2020, with newborn infants who were admitted to the neonatal unit in the first six hours after birth with suspicion of early-onset sepsis. The data from 104 cases that met the inclusion criteria were analysed in Fig. 1. Newborns with fetal distress in antenatal follow-up, with cord ABGA values below 7.0, a 5-min Apgar score below 5, cases for whom informed consent could not be obtained from the parents, and those with cardiac or metabolic disease were excluded from the study.

Ethics committee approval was obtained from Ethics Committee of the Cerrahpasa Faculty of Medicine, Istanbul University-Cerrahpasa (date: 05/12/21, approval no; 186107). Written informed consent from the parents was obtained for all participants.

Blood culture evaluation was performed in all cases before starting antibiotic treatment; following the guidelines of the Turkish neonatology society, lumbar puncture and chest X-ray were performed in necessary cases, and blood samples were taken into heparinized injectors and measured by ABL Flex (Radiometer) within 20 min^12^. Lactate and base excess (BE) in the cord ABGA and at the postnatal 6th-hour venous blood gas were registered. In addition, C-reactive protein (CRP) and procalcitonin (PCT) values of all cases were evaluated at the postnatal 24th hour. An algorithm was not determined for initiating antibiotics for the cases with EOS, and the decision to start antibiotic treatment in all cases was left to the consultant specialist neonatologist.

The threshold values of 15 mg/L for CRP, 2 ng/ml for PCT, − 10 mEq/L for blood gas BE were accepted according to EMA sepsis scoring, previous studies, and the limit values used by our hospital^4,7^. Threshold values of 2 and 4 mMol/L for lactate measures were determined relying on previous studies^8–10^.

The diagnosis of clinical sepsis was defined as having any relevant clinical findings and positive results for either CRP (> 15 mg/L) or PCT (2 ng/ml) at the postnatal 24th hour. Suspected sepsis was defined as whether there is a clinical symptom or not the presence of sepsis risk factors (chorioamnionitis, premature rupture of membranes above 18 h or above, maternal fever) in the newborns or clinical findings suggesting sepsis in follow-up without positive CRP or PCT results at the postnatal 24th hour's blood measures^11,12^. Laboratory findings are considered positive when CRP is above 15 mg/dl and/or PCT above 2 ng/ml. Clinical findings are irregular body temperature (> 38.5 °C or < 36 °C), cardiovascular instability (bradycardia or tachycardia and/or rhythm irregularity, oliguria (< 1 ml/kg/hour), hypotension, peripheral perfusion), skin and subcutaneous lesions (petechiae, sclerema), respiratory instability (apnea or tachypnea or increased oxygen demand or increased need for ventilation support), gastrointestinal (nutritional intolerance, insufficient breastfeeding, abdominal distention), and non-specific signs (irritability, lethargy, hypotonia) based on EMA score^7^.

Based on the postnatal 24th-hour evaluation, newborns were divided into clinical and suspected sepsis groups. First, the lactate and BE values in cord ABGA of the clinical sepsis group were compared to that of the suspected sepsis group. Then, the groups were compared regarding their lactate and BE values at the postnatal 6th hour shown in Fig. 1.

Fetal distress was accepted as abnormal cardiotocography detection in the regular antenatal follow-up of specialist obstetricians. Prelobor rupture of membranes (PROM) is defined as the rupture of membranes at least 18 h before the onset of labor^13^. Fever ≥ 39.0 °C once or 38.0–38.9 °C in two or more measurements 30 min apart with no other apparent source and one or more of the following: Except for accelerations, decelerations, and periods of significant variability, baseline fetal heart rate > 160 beats/min for ≥ 10 min, maternal white blood cell count > 15,000/mm^3^, in the absence of corticosteroids and ideally shifting to the left, purulent-appearing fluid from the cervical os seen by speculum examination^14^. Chorioamnionitis is defined as an acute inflammation of the membranes and chorion of the placenta, typically due to ascending polymicrobial bacterial infection in the setting of membrane rupture. Fetal growth restriction (FGR) is defined as the failure of the fetus to achieve its genetically determined growth potential^15^.

All statistical analyses were performed with R software (version 4.0.5) (R: A language and environment for statistical computing. R Foundation for Statistical Computing, Vienna, Austria, Available online: http/www.r-project.org/). Categorical variables were given as frequencies (percentage), and comparisons between groups were made by Pearson's Chi-squared test and Fisher's exact test. Continuous variables were analyzed with the Kolmogorov–Smirnov test, Shapiro–Wilk test, Q-Q plots, and histograms to check the normality assumption. Normally distributed variables were expressed as means ± standard deviations and compared with independent samples t-test. Non-normally distributed variables were shown as median (25–75th percentiles) and compared with the Mann–Whitney U test. pROC package was used to plot ROC curves (Receiver Operating Characteristic) and calculate sensitivity/specificity^16^. Optimal cut-off values were estimated according to Youden Index. *p* < 0.05 was considered significant.

### Ethics approval and consent to participate

An informed consent document with information about the study and in accordance with the requirements established by the “Ethics Committee of the Cerrahpasa Faculty of Medicine, Istanbul University-Cerrahpasa” was offered to the parents**.** Informed consent was obtained from parents. Ethics approval was obtained from the Ethics Committee of the Cerrahpasa Faculty of Medicine, Istanbul University-Cerrahpasa (date: 05/12/21, approval no; 186107) conducted in accordance with the Declaration of Helsinki.

### Consent to publish

Written informed consent was obtained from the parents.

## Results

All patients' mean gestational age, birth weight, and 5-min Apgar score were 34.1 ± 3.8 weeks, 2.368 ± 896 g, and 7.65 ± 1.30, respectively. Other patient characteristics are presented in Table 1. Bacterial growth was detected in 3 blood cultures, one in the suspected sepsis group (coagulase-negative *staphylococcus*) and two in the clinical sepsis group (*Escherichia coli*).

**Table 1:** Patient characteristics.

Characteristic	Overall (n = 104)^1^
Gestational age, wk	34.1 ± 3.8
Birth weight, g	2.368 ± 896
Type of delivery	
Vaginal delivery / C-Section	25 (24%) / 79 (76%)
Gender	
Female /male	43 (41%) / 61 (59%)
5-min Apgar score	7.65 ± 1.30
Umblical Cord pH	7,27 ± 0, 086
FGR	8 (7.7%)
Chorioamnionitis	4 (3.8%)
Urinary tract infection	6 (5.8%)
PROM (18 h or longer before birth)	16 (15%)
Maternal antibiotic treatment	7 (6.7%)

^1^Mean ± standard deviation; n (%).

*FGR* Fetal growth restriction, *PROM*, prelabor rupture of membranes.

The characteristics of the patients classified as clinical sepsis and suspected sepsis at the 24th hour are summarized in Table 2. No difference was observed between the two groups regarding gestational age, birth weight, type of delivery, gender, FGR, chorioamnionitis, urinary tract infection, PROM (18 h or longer before birth), and maternal antibiotic treatment. The 5-min Apgar score was lower in the clinical sepsis group (7 vs. 8, *p* = 0.0352).

**Table 2:** Comparison of patient characteristics of cases diagnosed with early-onset clinical sepsis and suspected sepsis.

Characteristic	Early-Onset sepsis		*P*-value
	Suspected sepsis n = 35 (33.7%)^1^	Clinical sepsis n = 69 (66.3%)^1^	
Gestational age, wk	35.0 (32.0–37.5)^1^	35.0 (33.0–37.0)^1^	0.793^2^
Birth weight, g	2,387 ± 892^1^	2,359 ± 904^1^	0.883^3^
Type of delivery			0.776^4^
Vaginal delivery	9 (26%)^1^	16 (23%)^1^	
C-Section	26 (74%)^1^	53 (77%)^1^	
Sex			0.843^4^
Female	14 (40%)^1^	29 (42%)^1^	
Male	21 (60%)^1^	40 (58%)^1^	
5-min Apgar score	8.00 (8.00–9.00)^1^	7.00 (7.00–9.00)^1^	**0.035**^2^
Umblical Cord pH	7.28 (7.24–7.32)^2^	7.29 (7.22–7.33)^2^	0.852^2^
FGR	2 (5.7%)^1^	6 (8.7%)^1^	0.714^5^
Chorioamnionitis	2 (5.7%)^1^	2 (2.9%)^1^	0.601^5^
Urinary tract infection	1 (2.9%)^1^	5 (7.2%)^1^	0.661^5^
PROM (18 h or longer before birth)	6 (17%)^1^	10 (14%)^1^	0.723^4^
Maternal antibiotic treatment	1 (2.9%)^1^	6 (8.7%)^1^	0.419^5^

^1^Median (25%-75%); Mean ± SD; n (%),^2^Mann-Whitney U test, ^3^Independent Sample t-test, ^4^Pearson's Chi-squared test, ^5^Fisher's exact test. Statistical significance was determined as *p* < 0.05.

*FGR* Fetal growth restriction, *PROM* Prelabor rupture of membranes.

Significant values are in [bold].

**Figure 2:**
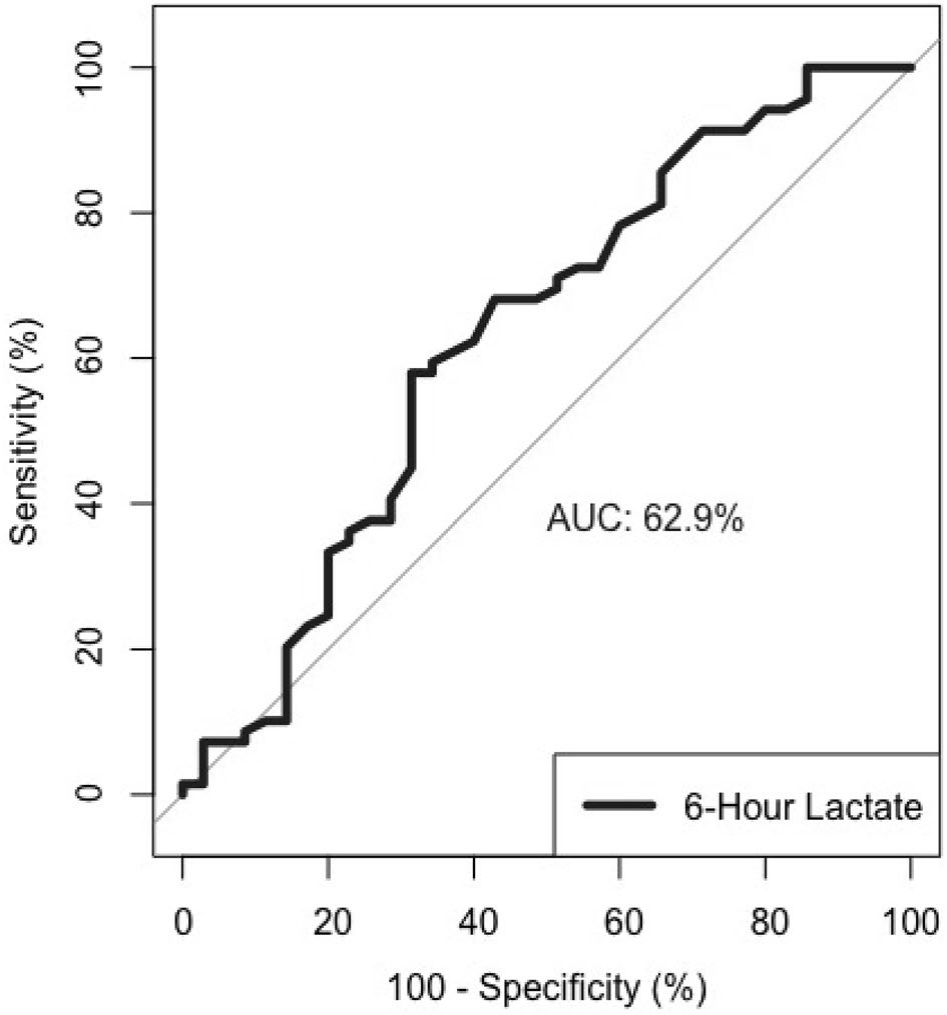
ROC curve for postnatal 6th-hour lactate value as an indicator of early onset clinical sepsis.

When the groups with the diagnosis of clinical sepsis and suspected sepsis were compared, clinical sepsis was more common in the cases with a lactate value above 2 mMol/L at the postnatal 6th-hour evaluation (*p* = 0.041). No association was found between the cord ABGA lactate, BE value, and the value of venous BE at the postnatal 6th hour and clinical sepsis in Table 3**.** The optimal 6th-hour lactate cut-off value, which identifies the cases diagnosed with clinical sepsis, was determined to be 3.38 mMol/L (sensitivity 57%, specificity 68%, *p* = 0.032) shown in Table 4, Fig. 2**.**

**Table 3:** Comparison of cord ABGA and postnatal 6th-hour evaluations in cases diagnosed with early-onset clinical sepsis and suspected sepsis.

	Early-onset sepsis		*P-*value
	Suspected sepsis, n = case/total (%)	Clinical sepsis, n = case/total (%)	
Cord ABGA			
Lactate (mMol/L)			
> 2	17/22 (77.3)	31/49 (63.2)	0.243^1^
< 2	5/22 (22.7)	18/49 (27.8)	
> 4	4/22 (18.2)	12/49 (24.5)	0.760^2^
< 4	18/22 (71.8)	37/49 (75.5)	
BE (mEq/L)			
> − 10	20/22 (90.9)	44/49 (89.7)	1^2^
< − 10	2/22 (9.1)	5/49 (10.3)	
Sixth hours results			
Lactate (mMol/L)			
> 2	28/35 (80)	65/69 (94.2)	**0.041**^**2**^
< 2	7/35 (20)	4/69 (5.8)	
> 4	8/35 (22.8)	24/69 (34.8)	0.213^1^
< 4	27/35 (77.2)	45/69 (65.2)	
BE (mEq/L)			
> − 10	33/35	64/69 (92.8)	1^2^
< − 10	2/35	5/69 (7.2)	

^1^Pearson's Chi-squared test, ^2^Fisher’s Exact Test. Statistical significance was determined as *p* < 0.05.

*ABGA* Arterial blood gas analysis, *BE* Base excess, *CRP* C-reactive protein, *PCT* Procalcitonin, *WBC* White blood cell.

Significant values are in [bold].

**Table 4:** Evaluation of the sensitivity and specificity of the postnatal cord ABGA (Postnatal 0th hour) and 6th-hour results in cases diagnosed with early-onset clinical sepsis.

Variable	Postnatal hours	Cut off levels	Sensitivity	Specificity	+ LR	− LR	PPV	NPV	Accuracy	AUC	*P-*value
Lactate (mMol/L)	0	> 1.45	91.84	9.09	1.01	0.90	69.2	33.3	66.2	0.492	0.916
		> 2	63.27	18.18	0.77	2.02	63.3	18.2	49.3		
		> 3.35*	38.78	77.27	1.71	0.79	79.2	36.2	50.7		
		> 4	24.49	81.82	1.35	0.92	75.0	32.7	42.3		
		> 6.25	12.24	95.45	2.69	0.92	85.7	32.8	38.0		
	6	> 2	94.20	20.00	1.18	0.29	69.9	63.6	69.2	0.629	**0.032**
		> 3.38*	57.97	68.57	1.84	0.61	78.4	45.3	61.5		
		> 4	36.23	77.14	1.59	0.83	75.8	38.0	50.0		
		> 6.55	8.70	91.43	1.01	1.00	66.7	33.7	36.5		
BE (mEq/L)	0	< − 1	81.63	4.55	0.86	4.04	65.6	10.0	57.7	0.466	0.645
		< − 2.7*	67.35	40.91	1.14	0.80	71.7	36.0	59.2		
		< − 10	10.20	90.91	1.12	0.99	71.4	31.3	35.2		
	6	< − 0.75	91.30	11.43	1.03	0.76	67.0	40.0	64.4	0.567	0.269
		< − 3.45*	57.97	62.86	1.56	0.67	75.5	43.1	59.6		
		< − 10	7.25	94.29	1.27	0.98	71.4	34.0	36.5		

*Optimal cut-off value, statistical significance was determined as *p* < 0.05.

*ABGA* Arterial blood gas analysis, *BE* Base excess, *CRP* C-reactive protein, *PCT* Procalcitonin, *WBC* White blood cell.

Significant values are in [bold].

The discrimination power between suspected and clinical early-onset neonatal sepsis in the postnatal 6th-hour CRP and PCT values was statistically insignificant (CRP; AUC = 0.567, *p* = 0.259 and PCT; AUC = 0.531, *p* = 0.599, respectively).

## Discussion

This study compared the cord ABGA and postnatal 6th-hour venous lactate and BE values of clinical and suspected sepsis cases. It was found that lactate value over 3.38 mMol/l (sensitivity 57%, specificity 68%, *p* = 0.032) measured at the postnatal 6th hour had a discriminative power on early-onset clinical sepsis. Lactate values are closely associated with morbidity and mortality since it is essential for final tissue oxygenation^17,18^. Therefore, when interpreting the acuity of lactate elevations in patients, it is crucial to determine the underlying cause of lacticemia and the associated prognosis. It is essential to exclude secondary causes of lacticemia (such as hypoxic-ischemic encephalopathy, necrotizing enterocolitis, and cyanotic congenital heart disease) in newborns with high lactate values^19^. Clinicians aim to detect and treat sepsis as early as possible, but no single or combined biomarker can provide this with 100 percent sensitivity and specificity^20^. Although a specificity higher than 85% is ideal for biomarkers, some authors find a specificity higher than 50% acceptable^21–23^. In our study, a lactate value above 3.38 mMol/L measured at the postnatal 6th hour was 68% specific for diagnosing clinical sepsis.

Rapid diagnosis and treatment of neonatal EOS are crucial to preventing morbidity and mortality. Clinical findings are often vague and non-specific, and commonly used biomarkers have low predictive values for early sepsis^5^. Although CRP is widely used to indicate bacterial sepsis in neonates and children, it may have several disadvantages, like a late response. CRP can generally be detected 12 h after the onset of clinical symptoms and reaches a plateau after 20–72 h^6^. It has been shown that the CRP level is high in cases diagnosed wit hypoxic ischemic encephalopathy and undergoing hypothermia treatment^24^. However, cases with suspected hypoxia were not included in our study, and the umbilical cord blood gas pH value and 5-min Apgar score of our cases show that there is no diagnosis neonatal asphyxia. Another frequently used parameter is PCT, which may increase earlier than CRP and reaches a peak in 6–8 h, remaining high for 24 h^6,25^. The measurement of lactate in blood gas results is faster, easier, and cheaper than these two frequently used parameters. In this study, lactate measurements at the 6th hour of life might be used to differentiate clinical sepsis from suspected sepsis.

Cord ABGA lactate was shown not to affect the differentiation of clinical sepsis and suspected sepsis in the diagnosis of EOS. Early-onset sepsis may be secondary pathogens from the mother's gastrointestinal or urinary flora by ascending the uterine compartment and infecting the amniotic fluid. Less commonly, it is caused by vertical transmission of bacteria from the mother's lower genital tract during vaginal delivery or by hematogenous spread through the placenta^26^. Sepsis is a systemic inflammatory response syndrome that develops secondary to an infection and is accompanied by circulatory dysfunction^27^. The following features of the placenta are well defined; It provides oxygen and nutrients to the fetus while removing carbon dioxide and other waste products, metabolizes several substances, and releases metabolic products into the maternal and/or fetal circulation, helping to protect the fetus against specific xenobiotic molecules, infections, and maternal diseases^28^. In addition, the protective effect of the placenta against cytokine release has been demonstrated in Covid 19 positive maternal placentas^29^. In our study, the absence of a correlation between lactate value in cord blood gas and clinical sepsis cases might be related to the protective effect of the placenta against the inflammatory process.

Cord ABGA and the postnatal 6th-hour BE measurements were shown not to affect the differentiation of clinical sepsis from suspected sepsis in the diagnosis of EOS. BE is primarily used to differentiate the metabolic or respiratory component of acidosis and affects the outcome mainly from the changes in blood bicarbonate level^30^. Base excess is not considered a sepsis biomarker by itself; it is mainly included in the EMA sepsis scoring system and is used to diagnose sepsis by demonstrating the presence of acidosis^7^. Similarly, our study showed that BE measurements do not differentiate between clinical and suspected sepsis.

The Apgar score quickly assesses the newborn's clinical status 1 and 5 min after birth. The Apgar score is obtained by evaluating five parameters (skin color, heart rate, reflexes, muscle tone, and respiratory activity). Apgar score was lower in the clinical sepsis group than in the suspected sepsis group in our study. In particular, a 5-min Apgar score below 7 indicates an increased risk of neonatal asphyxia^31^. Other objective indicators of antenatal hypoxia are cord blood gas pH, BE, and lactate levels^19,32,33^. In this study, no difference was observed between the two groups regarding cord blood gas lactate and BE values. This result is likely related to excluding cases with fetal distress from this study. It has been shown that a low Apgar score increases the risk of early-onset sepsis^12,34^. Similar to previous studies, a lower 5-min Apgar score was associated with clinical sepsis in our study.

The gold standard in diagnosing EOS is the presence of growth in culture from sterile fields (blood, urine, cerebrospinal fluid, pleura, peritoneum, and joint fluid)^4,23,35^. Our study observed a blood culture growth in two cases (1.9%) in the clinical sepsis group. Coagulase-negative *staphylococci* culture growth in the blood culture in the suspected sepsis group was considered more in favor of contamination. Initial clinical signs and biomarkers are not specific in EOS, which causes intensive use of antibiotics in suspected patients. The number of babies that need to be treated for 1 proven case of EOS in term and late preterm babies are reported in the literature to be between 40 and 100^36^. Studies have reported blood culture growth rates ranging from 0.1 to 40% in EOS^37^. This range is usually ascribed to an insufficient blood sample, maternal antibiotic use, and low value of bacteremia^6,20^. In a recent prospective multicenter study, a CRP value above 16 mg/L at 36 h and a procalcitonin value above 2.8 ng/L could distinguish it from culture-positive sepsis at a high sensitivity rate^38^. In addition, over time, EOS, which is caused by group B streptococci, decreased with the increase in the use of antenatal antibiotics, and gram-negative enteric bacteria (mainly Escherichia coli) became the leading factor in preterm infants^39,40^. Similarly, the number of proven sepsis was found to be relatively low in our study, and Escherichia coli was shown in the blood cultures of only two cases diagnosed with clinical sepsis. Therefore, using the threshold values of 15 mg/L for CRP and 2 ng/L for procalcitonin value is acceptable to differentiate clinical and suspected sepsis at the postnatal 24th hour in our study.

The main limitation of this study is the low number of culture-positive sepsis cases. The study's strength is the number of cases, the number of markers evaluated, and the fact that it was conducted with a homogeneous patient group with close laboratory follow-up.

In conclusion, the diagnosis of EOS and the duration of treatment remain controversial for non-culture-positive cases. Clinicians can use lactate levels as an easily measured laboratory test in the neonatal intensive care unit. Our study showed that the patients with high lactate value in the postnatal 6th-hour venous lactate value were associated with an early-onset clinical sepsis diagnosis. Therefore, clinicians should closely monitor cases with high lactate levels in the postnatal 6th hour for early diagnosis of neonatal sepsis.

## Data Availability

The datasets generated and/or analysed during the current study are not publicly available due to our hospital policy but are available from the corresponding author on reasonable request.
